# Case Report: Pediatric acute suppurative thyroiditis caused by *Eikenella corrodens*: a rare presentation revealing pyriform sinus fistula

**DOI:** 10.3389/fped.2025.1614566

**Published:** 2025-08-08

**Authors:** Fan Cui, Lin Zhao, Fanchao Dong, Zhiqiang Yang, Jianjun Dong

**Affiliations:** ^1^ Department of Endocrinology, Qilu Hospital of Shandong University, Jinan, Shandong, China; ^2^ Department of Endocrinology, Linyi Traditional Chinese Medicine Hospital, Linyi, Shandong, China; ^3^ Department of Nursing, Linyi Cancer Hospital, Linyi, Shandong, China; ^4^ Department of Breast Surgery, Linyi Cancer Hospital, Linyi, Shandong, China

**Keywords:** suppurative thyroiditis, subacute thyroiditis, *Eikenella corrodens*, pyriform sinus fistula, pediatric infection, branchial cleft anomaly

## Abstract

Acute suppurative thyroiditis (AST) is a rare, potentially life-threatening bacterial infection of the thyroid gland, characterized by rapid progression. We report a rare pediatric case of AST caused by *Eikenella corrodens*, secondary to a pyriform sinus fistula (PSF), initially misdiagnosed as subacute thyroiditis (SAT). A retrospective analysis highlights the diagnostic challenges and emphasizes the need for early suspicion of PSF in recurrent AST. Timely imaging (CT/laryngoscopy) and pathogen-directed antibiotics are critical to prevent life-threatening complications. This case underscores the importance of considering atypical pathogens and underlying anatomical anomalies in pediatric AST to guide targeted therapy.

## 1 Introduction

Acute suppurative thyroiditis (AST) is a rare but potentially life-threatening bacterial infection of the thyroid gland, particularly in children. It accounts for only 0.1%–0.7% of all thyroid disorders and is often misdiagnosed in its early phase due to nonspecific symptoms that mimic subacute thyroiditis (SAT), such as fever, anterior neck pain, and elevated inflammatory markers (1, 2). If not promptly diagnosed and treated, AST can progress rapidly and result in severe complications, with reported mortality rates ranging from 3.7% to 9% (3, 4).

In pediatric populations, AST is frequently associated with congenital anomalies of the branchial apparatus. Among these, pyriform sinus fistula (PSF—a remnant of the third or fourth branchial pouch—is the most common predisposing factor, accounting for over 90% of pediatric AST cases (5). The fistula serves as a conduit for oropharyngeal flora to reach the thyroid gland, which is normally resistant to infection due to its rich vascular and lymphatic supply, high iodine content, and encapsulation.

Although Gram-positive cocci such as *Staphylococcus aureus* and *Streptococcus pyogenes* are the most frequently isolated pathogens in AST, rare organisms have also been implicated (6). *Eikenella corrodens*, a facultative anaerobic Gram-negative bacillus that is part of the normal flora of the oropharynx and upper respiratory tract, is an exceptionally rare cause of AST. To date, only sporadic case reports have documented its role in thyroid infections, particularly in immunocompetent children (7). Its involvement presents unique diagnostic and therapeutic challenges due to its fastidious growth requirements and intrinsic resistance to commonly used antibiotics such as clindamycin and first-generation cephalosporins.

In this report, we present a rare case of pediatric AST caused by *Eikenella corrodens*, secondary to an underlying PSF. We describe how corticosteroid therapy initially masked the diagnosis, how targeted antibiotic adjustment led to resolution, and how timely imaging and surgical intervention were essential for definitive treatment. This case highlights the importance of considering both atypical pathogens and congenital anatomical defects in children with recurrent or refractory thyroiditis.

## 2 Case report

A 14-year-old male presented with a 1-week history of recurrent fever and left-sided neck pain that worsened with swallowing. He also reported a sore throat and cough 10 days prior to admission. He was initially evaluated in the otolaryngology department of a tertiary hospital, where flexible laryngoscopy revealed no significant abnormalities. However, due to palpable enlargement of the left thyroid lobe, a thyroid ultrasound was performed, which demonstrated a mixed echogenic area in the left lobe suggestive of SAT. The patient was subsequently referred to the endocrinology department for further evaluation.

Upon admission, laboratory investigations showed normal thyroid function and negative thyroid-specific antibodies. However, inflammatory markers were elevated: erythrocyte sedimentation rate (ESR) was 55 mm/h, white blood cell count (WBC) was 10.9 × 10⁹/L with 68% neutrophils, and C-reactive protein (CRP) was 70 mg/L. Based on these findings, SAT was diagnosed, and oral prednisolone (50 mg daily) was initiated, resulting in temporary symptomatic improvement.

**Figure 1:**
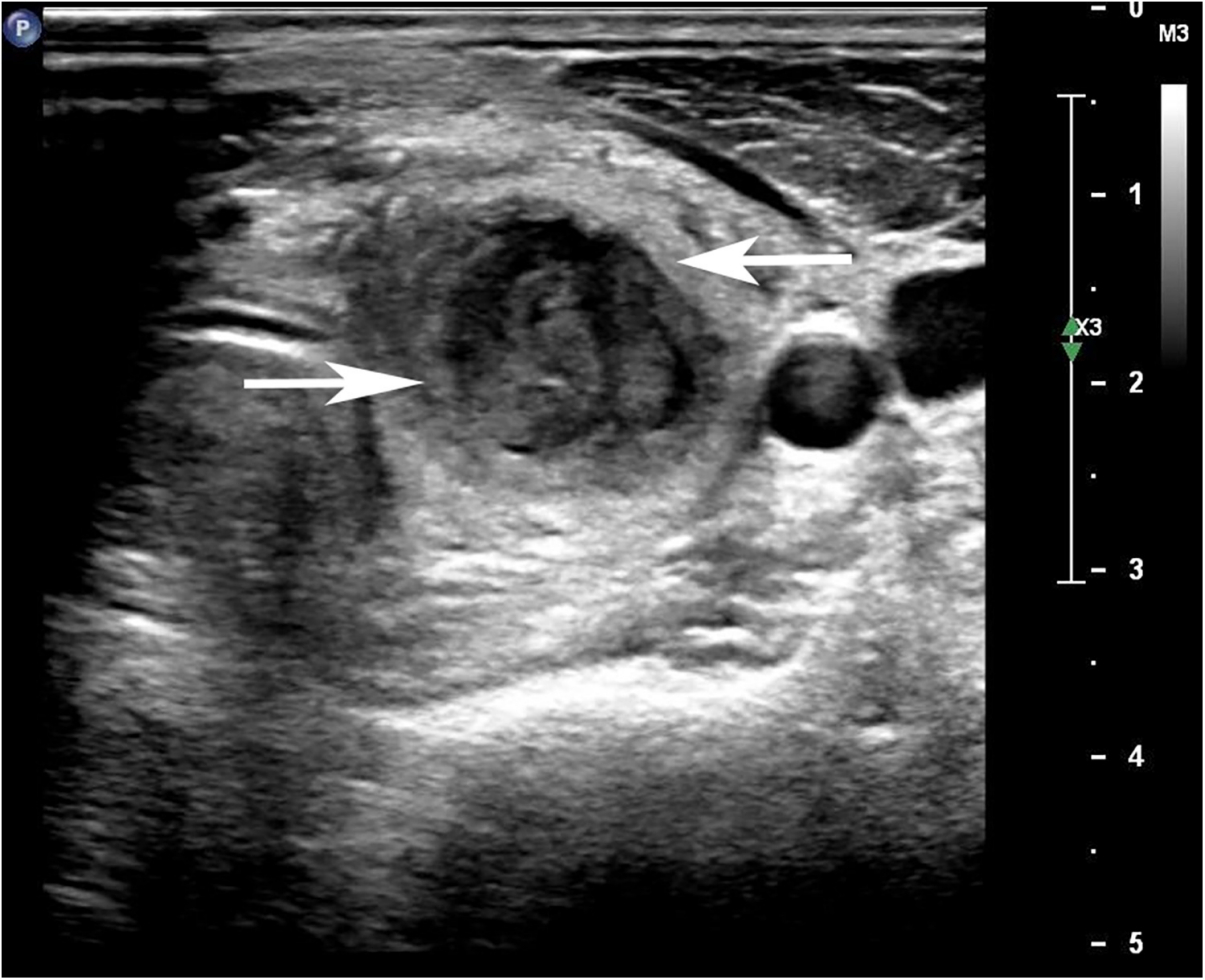
Ultrasound image of thyroid gland showing enlarged left lobe with ill-defined hypoechoic lesion with internal septations.

On hospital day 4, the patient developed worsening left-sided neck swelling, severe pain, and recurrent fever. Repeat laboratory tests showed increased inflammation: WBC 16.4 × 10⁹/L with 80% neutrophils, CRP 74.54 mg/L, and ESR 60 mm/h. Repeat thyroid ultrasound revealed a mixed echogenic mass in the left thyroid lobe with features suggestive of suppurative inflammation, along with associated left cervical lymphadenopathy (Figure 1). Ultrasound-guided fine-needle aspiration (FNA) yielded purulent yellow-green fluid, confirming a diagnosis of AST. Corticosteroids were promptly discontinued, and intravenous cefazolin (1 g every 8 h) combined with clindamycin (600 mg every 8 h) was initiated, as empirical therapy to cover common aerobic and anaerobic.

**Figure 2:**
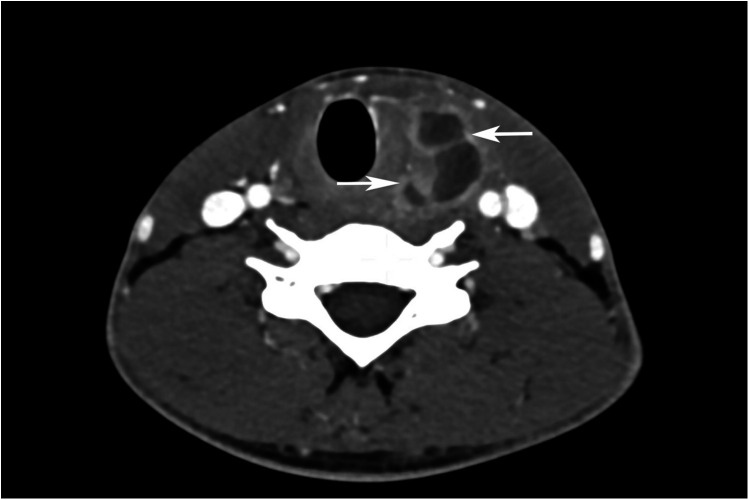
Contrast-enhanced axial section of thyroid gland shows enlarged left lobe with hypodense lesion with predominantly peripheral enhancement and few internal enhancing septae.

**Figure 3:**
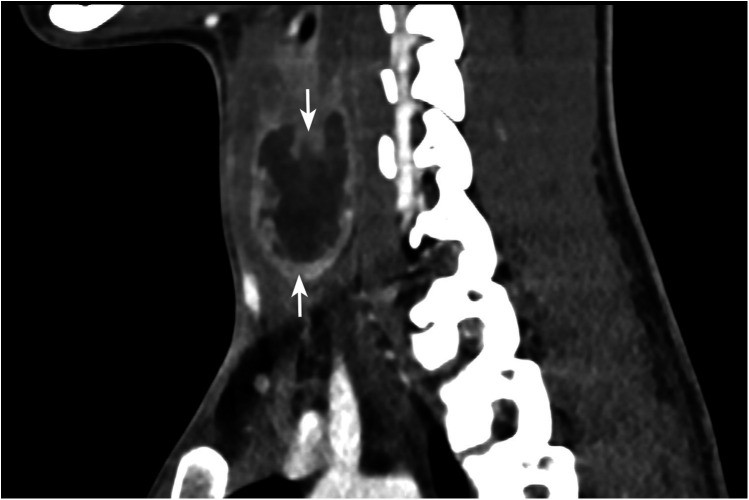
Contrast-enhanced coronal image of thyroid gland shows enlarged left lobe with hypodense lesion with predominantly peripheral enhancement and few internal enhancing septae.

Pus culture subsequently grew *Eikenella corrodens*, prompting escalation of antibiotic therapy to ceftriaxone (2 g once daily) based on susceptibility results, with continued drainage. The abscess was drained via ultrasound-guided percutaneous FNA (closed drainage), with repeated aspirations performed as needed. Due to persistent purulent discharge, contrast-enhanced neck computed tomography (CT) was performed. The CT scan demonstrated an abscess within the left thyroid lobe with extension into the surrounding soft tissue and retropharyngeal space (Figures 2, 3). Given the absence of known predisposing factors for AST, an underlying anatomical anomaly was suspected.

Repeat laryngoscopy revealed left pyriform sinus congestion with pseudomembrane formation, consistent with a PSF. The patient subsequently underwent cervical-approach excision of the fistulous tract with primary closure of the pyriform sinus opening under general anesthesia. Postoperative recovery was uneventful. At follow-up, the patient remained clinically well, with normalization of thyroid function and inflammatory markers. The patient and his family expressed relief and satisfaction after the definitive diagnosis and surgical treatment. They reported understanding of the management plan and expressed gratitude for the multidisciplinary care provided. To provide a clearer overview of the clinical course, key events, investigations, and interventions are summarized in Table 1.

**Table 1:** Summary of the clinical course.

Time point	Clinical events	Investigations	Interventions
Day –10 (symptom onset)	Patient developed sore throat and cough		
Day –7	Fever and left-sided neck pain began		
Day 0 (admission)	Admitted with neck swelling and fever	WBC ↑, CRP ↑, ESR↑; Thyroid US: hypoechoic lesion; laryngoscopy: no significant abnormalities	Oral prednisolone started
Day 4	Worsening neck pain and swelling, fever recurred	WBC ↑, CRP ↑ESR↑; Repeat US: mixed echogenic mass; FNA yielded pus	Steroid stopped; IV cefazolin + clindamycin; first percutaneous FNA drainage
Day 8	Persistent purulent discharge	Pus culture: Eikenella corrodens	Switched to ceftriaxone; repeated FNA drainage
	Suspected underlying anomaly	Contrast-enhanced CT: abscess in left lobe	
Day 10	Recurrent symptoms investigated	Laryngoscopy: PSF identified	Planned surgery
Day 14	Definitive treatment		Cervical approach excision of fistula with primary closure
Follow-up	Uneventful recovery	Thyroid labs normalized	No recurrence

US, ultrasound; FNA, fine-needle aspiration; PSF, pyriform sinus fistula; CRP, C-reactive protein; ESR, erythrocyte sedimentation rate; WBC, white blood cell count; IV, intravenous.

## 3 Discussion

AST in children requires prompt diagnosis, empirical broad-spectrum antibiotic therapy, and timely abscess drainage. In the early phase, AST often mimics SAT, as both conditions can present with fever, neck pain, and elevated white blood cell counts while maintaining normal thyroid function (5). Imaging findings in early AST, such as hypoechoic areas on ultrasound or low-density lesions on CT, may further resemble SAT. Misdiagnosis may lead to inappropriate corticosteroid therapy, which can suppress inflammation while allowing infection to progress.

In our case, the patient initially responded to corticosteroids with temporary symptom relief, which likely masked the underlying suppurative infection. However, the subsequent rise in inflammatory markers and worsening clinical status prompted reevaluation. This highlights the importance of maintaining clinical vigilance when managing presumed SAT, particularly in pediatric patients. When the diagnosis is unclear, FNA should be performed to identify purulent material or obtain cultures (1, 8). Even in the absence of obvious fluid collections, FNA washout may yield diagnostic evidence of infection. In our case, a combination of ultrasound-guided FNA and contrast-enhanced CT established the diagnosis of AST.

AST is typically caused by oral flora, with gram-positive cocci being the most common pathogen (5). However, anaerobic and polymicrobial infections occur in up to 30% of cases (9). Therefore, empirical antibiotic regimens should thus cover both aerobic and anaerobic organisms. First-line agents include penicillin and clindamycin (6), although resistance must be considered. In our case, empirical use of clindamycin initially showed poor results. Considering the possibility of resistant strains, the collected pus culture revealed *Eikenella corrodens—*a facultative anaerobic gram-negative bacillus that rarely causes thyroid infections.

*Eikenella corrodens* is part of the normal flora of the oral cavity, upper respiratory tract, gastrointestinal tract, and genitourinary tract. It can act as an opportunistic pathogen, especially in immunocompromised hosts, such as those with diabetes, cancer, HIV, or oral injuries, and is known to cause deep-seated abscesses (10). It is generally susceptible to ampicillin and chloramphenicol but resistant to first-generation cephalosporins and clindamycin (11). In our patient, fever resolution following appropriate antibiotic adjustment and drainage highlights the importance of tailoring therapy based on culture results. This underscores the importance of tailoring antibiotic therapy based on accurate pathogen identification. For bacteria like *Eikenella corrodens*, which require specific culture conditions and longer incubation times, we must learn from experience. When some broad-spectrum antibiotic treatment is ineffective, considering the presence of rare opportunistic pathogens and promptly adjusting antibiotic regimens is crucial to preventing infection exacerbation. The effectiveness of adjusted antibiotic therapy in this case is a testament to this approach.

Despite initial improvement, the patient experienced recurrent neck abscesses, prompting further investigation into the underlying etiology. Thyroid abscess in children is a rare complication of AST. This is primarily due to the thyroid's encapsulation, high iodine concentration, rich lymphatic drainage, and dual blood supply, which confer strong resistance to infection, unless underlying anomalies are present, acute inflammation is unlikely to occur (5, 12). Microorganisms found in pediatric AST are typically part of the normal oropharyngeal flora. In children with normal immune function, local upper respiratory tract flora can spread to the perithyroidal space and thyroid through fistulas (13). These fistulas include thyroglossal duct remnants, preexisting thyroid disease, or trauma, with the most common factor being PSF, which is a remnant of the third and fourth branchial pouches (12). Considering the absence of underlying thyroid disease and trauma in this case, the susceptibility to AST may be due to the presence of embryonic remnants of the third or fourth branchial cleft.

From an embryological perspective, the third and fourth branchial clefts fuse to form the pyriform sinus, extending towards the midline near the thyroid gland. The fistula may reach the thyroid surface or terminate within the thyroid. Due to asymmetry in embryonic development, the fistula predominantly appears on the left side (14). Due to its rarity, the diagnosis of PSF is often overlooked, leading to recurrent cervical abscesses and delayed treatment. The “gold standard” for diagnosing PSF is electronic laryngoscopy. Additionally, barium swallow, ultrasound, and CT scans are helpful in diagnosing this condition (15, 16). In this case, the child presented with AST as the initial symptom, with infection involving the left thyroid lobe, and was eventually diagnosed with PSF via follow-up laryngoscopy. Early recognition was hindered due to the rarity of the condition, limited clinical experience, and the stage of disease progression. In the early stages, atypical symptoms can lead to misdiagnosis as SAT. During the acute inflammatory phase, the fistula may be secondarily obstructed by edema, making it difficult to diagnose the fistula via laryngoscopy or esophagography, resulting in missed diagnoses. Therefore, esophagography should be performed during the resting phase. This suggests that for patients with recurrent AST, it is necessary to repeat esophagography to reassess anatomical defects related to thyroiditis or neck infections to identify PSF.

The classic treatment method involves excising the fistula via a cervical approach, possibly with or without thyroid lobectomy (17). However, less invasive alternative approaches have been developed, reducing the risk of complications along the fistula tract, such as endoscopic electrocauterization (18). In this case, after actively controlling the inflammation, the patient underwent pyriform sinus fistula closure surgery under general anesthesia with good wound healing and no recurrence during follow-up.

This case also illustrates both the strengths and limitations of the diagnostic and therapeutic approaches adopted. The strengths of this case include early use of ultrasound-guided FNA for microbiological confirmation, timely adjustment of antibiotic therapy based on culture results, and definitive surgical correction of the underlying PSF, which together ensured a good outcome. However, as a single-case experience, it has inherent limitations. The rarity of *Eikenella corrodens* (a typically commensal oral organism) in AST and the difficulty in visualizing the fistula during the acute phase may limit generalizability. Furthermore, diagnostic challenges due to overlapping features with SAT can delay recognition. Similar findings were reported in the case series by Nicoucar et al., where PSF was identified as the underlying cause of recurrent AST (15).

In conclusion, AST in children, though rare, should be suspected when standard SAT treatment fails to yield sustained improvement. Caution is warranted with corticosteroid use in ambiguous cases. FNA is essential for early differentiation, and culture-directed antibiotic adjustment is critical, especially when rare pathogens like *Eikenella corrodens* are involved. Finally, recurrent AST should prompt evaluation for congenital anomalies such as PSF, with surgical correction necessary to prevent recurrence and achieve definitive cure.

## Data Availability

The original contributions presented in the study are included in the article/Supplementary Material, further inquiries can be directed to the corresponding author.
